# Postdoctoral Researchers in the UK: A Snapshot at Factors Affecting Their Research Output

**DOI:** 10.1371/journal.pone.0093890

**Published:** 2014-04-04

**Authors:** Fatima M. Felisberti, Rebecca Sear

**Affiliations:** 1 Kingston University, Psychology Department, London, United Kingdom; 2 London School of Hygiene and Tropical Medicine, Department of Population Health, London, United Kingdom; Université de Montréal, Canada

## Abstract

Postdoctoral training is a typical step in the course of an academic career, but very little is known about postdoctoral researchers (PDRs) working in the UK. This study used an online survey to explore, for the first time, relevant environmental factors which may be linked to the research output of PDRs in terms of the number of peer-reviewed articles per year of PDR employment. The findings showed reliable links between the research output and research institutions, time spent as PDR, and parental education, whereas no clear links were observed between PDRs' output and research area, nationality, gender, number of siblings, or work environment. PDRs based in universities tended to publish, on average, more than the ones based in research centres. PDRs with children tended to stay longer in postdoctoral employment than PDRs without children. Moreover, research output tended to be higher in PDRs with fathers educated at secondary or higher level. The work environment did not affect output directly, but about 1/5 of PDRs were not satisfied with their job or institutional support and about 2/3 of them perceived their job prospects as “difficult”. The results from this exploratory study raise important questions, which need to be addressed in large-scale studies in order to understand (and monitor) how PDRs' family and work environment interact with their research output—an essential step given the crucial role of PDRs in research and development in the country.

## Introduction

Postdoctoral researchers (PDRs, also referred to as *postdocs*) have been an integral part of European academia for centuries, and the model was exported to the USA in the 1870s [1]. Originally, PDRs worked in relatively large-scale laboratory-based projects, mainly in the biological sciences. With the expansion and the diversification of research in science and technology, the range of disciplines offering postdoctoral training increased dramatically. Nowadays postdoctoral training is a typical step in academic career progression. However, a report released by the Council for Science and Technology (CST, 2007) raised a number of concerns about the quality of postdoctoral training available in the UK and called for a radical overhaul of the way the university system treats young PDRs; the lack of a clear career path, associated with job insecurity, was one of PDRs main concerns. The report's authors recommended addressing the high levels of dissatisfaction and distress among UK early-career researchers to strengthen future research and related investments in the country. Some of the problems may be related to the decline in the proportion of European and North American postgraduates opting for postdoctoral training [2], but more studies are needed to confirm such findings.

In a report for the UK Institute for Employment Studies (IES, 2002) Sir Gareth Roberts referred to the lack of effort to deal with the issues faced by young researchers [3], which was echoed in other countries [4]. However, there is no compiled and updated information about the PDRs experience easily available to early-career researchers. To make the task more complex, PDRs in the UK are employed for the same post under different names: research assistants, research associates, research scientists, research fellows, postdoctoral assistants, postdoctoral associates, or postdoctoral fellows (e.g. http://www.jobs.ac.uk). Frequently, the first three posts also may include students who have not completed their PhD dissertations. Many people outside academia do not know the difference between PhD students and PDRs (who already have a PhD degree) and refer to them simply as “postgraduate students”.

The majority of PDRs choose a postdoctoral appointment to learn and/or hone complex research skills, to carry out research in a new and vital area, and/or to increase their research output in order to improve their chances when competing for tenured positions in academia [5]–[8] (the terms research “output” and “productivity” are used interchangeably in this study). The research output—typically measured as the number of peer-reviewed publications—is crucial to PDRs' career progression. There are very few studies about the factors that modulate PDRs' output, given that prospective employers tend to use it as an indicator of intellectual ability and/or productivity.

According to Gati (1990), the quality of career decisions under uncertainty is affected by how decisions are framed. Since the decision to embark on an academic career is full of uncertainties [9], it is essential to identify some of the key factors which may influence the process positively as well as to identify the factors which may be linked with negative effects. Here we investigate whether demographic factors, family and work environment are associated with PDRs productivity and job satisfaction.

Previous research suggests that the level of support and mentoring available to PDRs in research institutions seems to affect their output [10], [11]. Anecdotally, the PDRs' work experience in the UK may be difficult: the salary structure does not progress beyond a couple of years, there are rare opportunities to work on a rolling contract, the number of new tenured positions is limited, and the working hours are long [12].

Gender is another factor that seems to affect career progress in academia. Previous studies have shown marked differences in the career choices of men and women [13]–[15]. For many women, the time to make fertility decisions frequently coincides with the start of their postdoctoral training (mid-20s to early 30s), which is one of the reasons believed to be behind the gender gap in science [16]. Indeed, in many surveys about male and female academics, childcare was seen as a major barrier to professional progression [17]. Interestingly, both groups were virtually unanimous in believing that it is extremely difficult for a woman to advance in the area *and* have a family.

A few previous surveys, mainly outside the UK, involving PDRs working in Chemistry, Physics and Mathematical Sciences showed that compared to men, women in Biochemistry stayed longer as PDRs and advanced more slowly to tenure-track faculty positions [18], [19]. Worse still, a large number of female PDRs in Mathematics failed to reach a tenured position [20]. Lengthy periods of postdoctoral employment may influence subsequent career decisions, as a report commissioned by the Institute of Physics and the Royal Society of Chemistry revealed a significant (and negative) association between the number of years as a PDR and the desire to pursue an academic career in women, but not in men [8].

As well as their own family decisions, it is feasible that PDRs' career progression may be influenced by their family of origin: family-related factors have been shown to make critical contributions to a student's performance, which may affect subsequent success in academia [21]. For example, it is known that the parents' involvement in a child's learning led to higher academic achievements [21], [22]. Moreover, family size and birth order were linked to some measures of intelligence in large surveys with young adults (19 years-old) and children (7–12 years-old) [23], [24]. No study to date has investigated if the PDRs' parental education, family size, and birth order were linked to their productivity.

This study explored how the research output (in terms of the number of peer-reviewed articles published per year of postdoctoral employment) was associated with environmental variables using data from a survey of PDRs based in UK institutions. Due to the exploratory nature of this study, broad hypothesis were formulated: (i) the output—i.e. productivity—of PDRs with children (especially females) would be more affected than PDRs without children, due to time constraints imposed by child-rearing, and (ii) PDRs' job satisfaction and institutional support would be positively correlated with their output. The information obtained here could guide future large-scale studies and help to develop and/or implement policies and programmes to support PDRs in a crucial step in the early-years of their scientific career.

## Methods

This study was approved by the Psychology Ethics committee, and followed the guidelines set by the Faculty Ethics Committee, at Kingston University, in agreement with the British Psychological Society. A consent form was displayed before the questions appeared on a computer screen and consent was implicit if participants proceeded to the online questionnaire.

### Participants

Participants were recruited via an invitation posted in Vitae website (http://www.vitae.ac.uk) and by emails sent to heads of departments in England, Wales, Scotland and Northern Ireland (mainly in the fields of Psychology and Life Sciences). Half of the invitations were sent to post-92 universities and half to pre-92 universities (see below). Participants who had completed the questionnaire were then asked if they wanted to make the online link to the study available to other PDRs they knew (i.e. by word of mouth, also referred to as “snowballing” procedure).

Entries with the same IP addresses and/or email addresses were eliminated from the sample prior data analysis. The initial sample had 282 participants, but 84 participants completed only up to the 10 initial demographic related questions and could not be included in the study due to the lack of data about their publications.

The study sample containing research productivity data (*N* = 198; 78 males, 120 females) included PDRs employed in research institutions and pre- and post-1992 UK universities (the latter are former polytechnics, central institutions or colleges of higher education who were given university status through the Further and Higher Education Act in 1992) (Table S1). Ten participants were removed from the study; they were either lecturers still working part-time as research assistants (*N* = 3) or outliers in terms of length of PDR experience and publication record (PDRs who had published 15 or more articles over a period longer than 10 years (*N* = 7)). The final number of PDRs was 188 (72 males, 116 females). The age of most PDRs was in the 26–40 years range, with 14 PDRs over 40 years old. It is worth noting that a few respondents did not answer all the questions, which was reflected in the different degrees of freedom reported in the results section.

### Procedure

The online questionnaire was available online from Feb/2008 to Feb/2009 (SurveyMonkey website, www.surveymonkey.com). The questionnaire consisted of a set of questions related to different aspects of PDRs' life (e.g. demographic data, work environment, psychometric tests). The data related to psychometrics tests will be analysed in a separate report. The completion of the online questions took between 20–40 min. The questions were broadly grouped under the following headings (for more details see Table S2):

- Demographic data
- Job description, type and location
- Work environment
- Research output
- Family-related factors

The number of book chapters, books, and peer-reviewed conference abstracts was also collected, but not included in the final output because it was not possible to equate that type of output to articles published in peer-reviewed journals.

### Data sampling

One of the weaknesses with the sampling method used in this study was that participation was voluntary and therefore this cannot be considered a flawless representative sample. For example, PDRs filling in the questionnaire might have been particularly happy or particularly unhappy with their PDR experience. Nonetheless, this sample had a wide range of research institutions and universities distributed across different regions in the UK, including some of the most productive in terms of number of publications, number of registered patents, and volume of research funding.

A second point to consider was the division of research areas into three main groups: Life Sciences, Social Sciences, and Physics, Chemistry and Mathematical Sciences (PCMS). The first group included PDRs in biological and medical research and the second group included PDRs in psychological and social science research (mostly Psychology), whereas the third group of PDRs was much more diverse. We used this grouping because there are disciplinary differences in postdoctoral training, which have been recognised in previous studies. Those studies have addressed many issues related to PDRs in Chemistry, Physics and Engineering, but there has been less research in the other disciplinary areas (see introduction). Additionally, some universities and professional societies in the UK have programs or discussion groups tailored to support PDRs working in PCMS, which is not common in Life Sciences and Social Sciences. We therefore consider the grouping used here is worthwhile for comparing PDRs in the Life and Social Sciences with those in the ‘hard’ sciences, but caution that—given the heterogeneity within each group—some of the comparisons between the three groups should be used primarily as guidance for future studies with larger samples.

Our outcome measure is a measure of research output—the number of peer-reviewed publications per year of postdoctoral employment—but does not take into account any measure of ‘quality’ of these publications. Information on the journals in which the participants published their work could not be directly accessed, as the questionnaires were filled anonymously. Therefore, the research output could be not linked to the impact factor of the publications. If such analysis is considered useful, then this problem could be solved in the near future with open databases specific for the publications of PDRs in universities and research centres.

### Data analysis

The study employed the Chi-Square test and repeated-measures analyses of covariance (ANCOVA), sometimes referred to as univariate ANOVA.

The PDRs' productivity (measured as the number of peer-reviewed articles/time spent as PDR) was the dependent variable and had gender as a weighting factor. Because the data were not normally distributed – there was a peak of 0 publications, particularly driven by those with <2 years PD experience - PDRs in their first year of employment with either a book chapter and/or peer-reviewed conference presentations (but no peer-reviewed journal articles) were re-classified as having “0.05 publications/time spent as PDR”; whereas PDRs employed for ≥2 years and without any peer-reviewed articles were left with “zero publications”. The normal distribution of the square-rooted output had a small negative skewness. The z for skewness was -1, which is considered an acceptable value for a normal distribution [25], [26]. The output was then reverted to raw values in the reported results.

The variables “research institutions” (i.e. work place) and “research area” were used as covariate factors in the data analysis, except when they were the independent variable analysed. The statistical analysis was also weighted by the PDRs gender. All pair wise comparisons were carried out using Bonferroni adjustments. The data was analysed taking into account full-time and part-time PDRs. The results of the statistical analysis of part-time and full-time PDRs are given separately only when there were significant differences between them; otherwise, the results refer to all PDRs.

Partial eta-squared (*pη^2^*) refers to the effect size, and the cut-off values suggested by Cohen [27], [28] are: 0.01 small, 0.06 medium, and 0.14 large. The *pη^2^* can be transformed into a ƒ value (a more familiar index for some) using the software G*Power. Accordingly, the cut-off values for ƒ are: 0.10 small, 0.25 medium, and 0.40 large. Note that some researchers consider such benchmarks to be “rules of thumb”—arbitrary values and recommend caution with their interpretation [29].

## Results

The PDRs in this study referred to their position as research fellows (44%), research associates (40%), research assistants (7%), postdoctoral scientists (3%), postdoctoral researchers (3%), or research officers (3%). The “research fellow” position seems to be considered by many PDRs as better paid and more demanding, whereas the “research officer” position tends to involve a longer-term employment. However, the reasons for the different titles for the same postdoctoral positions are not clear, nor are the criteria used to define them.

As previously mentioned, the statistical analysis had gender as a weighting factor and the variables “research institution” and “research area” as covariate factors, except when they were the independent variables analysed.

### Research area

The research areas were aggregated in three main groups: Life Sciences, Social Sciences, and Physics, Chemistry and Mathematical Sciences (PCMS).

A Chi-Square test of independence was used to examine the relation between research area and PDR's gender. The relation between these variables was significant (χ^2^ (2, *N* = 188) = 8.23, *p*<.02). Slightly more female PDRs (33%) worked in Social Sciences than males (22%), whereas males (31%) were more frequent than females (14%) in PCMS. Nonetheless, most male (47%) and female (54%) PDRs in this study were working in Life Sciences.

An ANCOVA showed that the output varied significantly with the research area (*F*(2, 183) = 3.25, *p* = .04, *pη^2^* = .03, ƒ = .19). The research output in Social Sciences and PCMS was similar, but output in Social Sciences was significantly higher than in Life Sciences (*p* = .05) (Table 1). The difference in output might have been driven by PDRs in their first two years of employment in Social Sciences, who had an output of 0.82, against outputs of 0.22 in Life Sciences and 0.36 in PMCS. Caution is needed, however, since the effect size was small. In addition, when the work place was used as a covariate factor, the relationship between output and research area turned statistically non-significant (*F*(2, 184) = 1.84, *p* = .16). As expected, planned contrast showed that PDRs' output was significantly related to time spent as PDR (*t*(183) = 4.83, *p*<.001, *r* = .33).

**Table 1:** PDR's research output according to research area and research institution: mean ± standard error and 95% confidence interval (brackets).

	**Social Sciences (*N* = 52)**
Research Centres (*N* = 39)	n/a
Russell Group Universities (*N* = 99)	0.9±0.1 [0.6, 1.1]
Other Universities (*N* = 47)	1.1±0.1 [0.8, 1.3]
	**Life Sciences (*N* = 96)**
Research Centres	0.5±0.1 [0.2, 0.7]
Russell Group Universities	0.7±0.1 [0.5, 0.9]
Other Universities	0.8±0.2 [0.3, 1.2]
	**PCMS (*N* = 37)**
Research Centres	0.5±0.3 [0, 1.1]
Russell Group Universities	1.0±0.2 [0.7, 1.4]
Other Universities	1.1±0.2 [0.7, 1.5]

“Other Sciences” refers to Physics, Chemistry and Mathematical Sciences (PCMS). *N* indicates the sample size.

### Research institutions

The PDRs in this study developed their research work in research centres or universities. The universities were subdivided into two groups: universities in the Russell group (http://www.russellgroup.ac.uk), considered to be the most research intensive UK universities, and “other universities”, most of them referred to as “post-92 universities” since they were given the status of universities through the Further and Higher Education Act 1992 (http://en.wikipedia.org/wiki/New_universities, accessed 2014 Mar 23).

An ANCOVA confirmed the link between the work place and PDRs' output (*F*(2, 184) = 6.13, *p* = .003, *pη^2^* = .06, ƒ = .26), both indicated a medium effect size. The covariate, research area, was not significantly related to the output by work place (*F*<1). Planned contrast showed that PDRs' output in universities was similar (*t*(184) = −1.72, *p* = .09, *r* = .13) independently of whether they were in the Russell group universities or not. On the other hand, the output was significantly higher in universities than in research centres (*t*(184) = −3.49, *p* = .001, *r* = .25) (Table 1).

### Nationality

We investigated whether British PDRs differed in productivity from non-British PDRs, and also ran further tests on nationality when aggregated in four main groups: British, non-British Europeans, North Americans, and “Other nationalities”.

Exploratory analyses showed that British and non-British PDRs tended to congregate in different research institutions and areas. A Chi-square test showed that a significant relation between research institution and PDR's nationality (χ^2^ (6, *N* = 185) = 21.54, *p*<.001). Most British PDRs worked in universities in the Russell group (57% of the British sample) and in post-92 universities (34%), whereas most non-British PDRs (33% of the non-British sample) were working in research centres or in Russell group universities (49%). Another significant relation was found between research area and the PDR's nationality (four groups: χ^2^ (6, *N* = 185) = 19.80, *p* = .003), but not when the sample was divided into British vs. non-British PDRs (χ^2^ (2, *N* = 185) = 4.43, *p* = .11). In short, 43% of the British PDRs worked in Life Sciences and 34% in Social Sciences, whereas 59% of the non-British PDRs worked in Life Sciences and 44% in Social Sciences.

The output of British and non-British PDRs was statistically similar (*F*(1, 181) = 3.37, *p* = .07), despite a higher 95% confidence interval for British and North American PDRs (Table 2).

**Table 2:** PDRs research output according to their nationality: mean ± standard error and 95% confidence interval (brackets).

	Research Output
British (*N* = 83)	0.9±0.1 [0.8, 1.1]
non-British (*N* = 102)	0.7±0.1 [0.5, 0.8]
---------------------------------------	
British (*N* = 83)	0.9±0.1 [0.8, 1.1]
non-British Europeans (*N* = 73)	0.6±0.1 [0.5, 0.8]
North Americans (*N* = 11)	1.0±0.2 [0.6, 1.5]
Other Nationalities (*N* = 18)	0.6±0.2 [0.3, 0.9]

*N* indicates the sample size.

### Time spent as PDR

An exploratory analysis showed some differences between PDRs with and without children. PDRs with children had spent more time in postdoctoral work than PDRs without children (Chi-Square test: χ^2^ (3, *N* = 185) = 30.64, *p*<.001).

As expected the longer the time sent as PDR, the higher the productivity per year (*F*(3, 182) = 7.82, *p*<.001, *pη^2^* = .11, ƒ = .35), with a medium effect size. The output in the first two years of PDR employment was statistically lower than the output for up to 3 years (*p* = .04), 5 years (*p* = .03) and 6 years (*p*<.001), whereas the output for 3 or more years spent as PDRs was similar (Table 3).

**Table 3:** PDRs research output in relation to the duration of postdoctoral employment: mean ± standard error and 95% confidence interval (brackets).

	Time as PDR
Up to 2 years (*N* = 69)	0.5±0.1 [0.3, 0.7]
Up to 3 years (*N* = 36)	0.9±0.1 [0.7, 1.1]
Up to 5 years (*N* = 51)	1.0±0.1 [0.8, 1.1]
Up to 6 years (*N* = 32)	1.1±0.1 [0.8, 1.3]

*N* indicates the sample size.

### Gender and children

From the 185 PDRs who answered this question 148 did not have children and 37 had one or more children (one child  = 17, two children  = 13, three children  = 7). Of the 14 part-time PDRs, 12 were females and more than half (*N* = 7) of them had children.

There were no gender differences in overall PDRs' output (*F*<1). At first sight, PDRs with children tended to publish more than PDRs without children (*F*(1, 185) = 4.11, *p* = .04, *pη^2^* = .02, ƒ = .14). A more detailed analysis, however, revealed that the difference between PDRs with and without children was not significant with the covariate factors (*F*(1, 183) = 2.22, *p* = .14) (Table 4).

**Table 4:** PDRs research output in relation to the presence or absence of children: mean ± standard error and 95% confidence interval (brackets).

	Males (*N* = 71)	Females (*N* = 116)
with Children (*N* = 37)	1.0±0.2 [0.6, 1.5]	0.8±0.1 [0.5, 1.1]
no Children (*N* = 148)	0.8±0.1 [0.6, 1.0]	0.8±0.2 [0.6, 0.9]

*N* indicates the sample size.

Although part-time work may play an important role in helping PDRs with children to increase their research output, the analysis with the part-time/full-time factor added as covariate did not reveal a reliable association (*F*(1, 182) = 2.49, *p* = .12). Furthermore, there were no significant interactions between gender and output, but male PDRs with children tended to publish slightly more than females with children, whereas publications by male and female PDRs without children was identical.

A larger sample of PDRs with children is necessary to confirm the current observation in order to safely rule out a Type II error.

### Family factors

#### Number of siblings and birth order

The PDRs' number of siblings varied from none (*N* = 20) to one (*N* = 93), two (*N* = 45) or three or more (*N* = 30). There was no significant association between research output and PDRs' number of siblings (*F*(3, 182) = 1.33, *p* = .27). Likewise, no link between research output and birth order was observed, independently of whether birth order was analysed as separate categories (i.e. from first-born up to fifth-(or higher order)born) (*F*(4, 179) = 1.10, *p* = .36) or simply dichotomised as first-born or not (*F*<1).

#### Parents' education

The educational level of the PDRs' parents was aggregated into four groups, and the relationship between such groups and productivity was analysed. The grouping of the parents was made according to the highest educational level attained (*m* refers to mother and *f* refers to father): primary education (*N_mf_* = 16), secondary education (*N_m_* = 85; *N_f_* = 60), university degree (*N_m_* = 54; *N_f_* = 62), and postgraduate degree (*N_m_* = 34; *N_f_* = 51).

PDRs with parents who had completed only primary education tended to have a lower research output than the PDRs with both parents with secondary or a higher education level, but such differences were not significant (*F*(3, 182) = 1.28, *p* = .28) (Table 5). Significant findings emerged, however, when the education level of the father and the mother were analysed separately (Table 5). The output varied with the education level of PDRs' fathers (*F*(3, 182) = 2.84, *p* = .04, *pη^2^* = .05, ƒ = .20), but not with the education level of their mothers (*F*<1).

**Table 5:** PDRs research output in relation to their parents education: mean ± standard error and 95% confidence interval (brackets).

*Education Level*	Father (N = 188)	Mother (N = 188)
Primary	0.5±0.1 [0.2, 0.7]	0.5±0.1 [0.2, 0.7]
Secondary	0.8±0.1 [0.7, 1.0]	0.8±0.1 [0.7, 0.9]
University	0.7±0.1 [0.6, 0.8]	0.8±0.1 [0.7, 0.9]
Postgraduate	0.8±0.1 [0.6, 0.9]	0.7±0.1 [0.6, 0.9]

*N* indicates the sample size.

### Work environment

A summary of the questions related to the work environment are available as supplementary material. Note that a few PDRs didn't answer all the questions (i.e. variable *df*).

#### Institutional support

**Figure 1:**
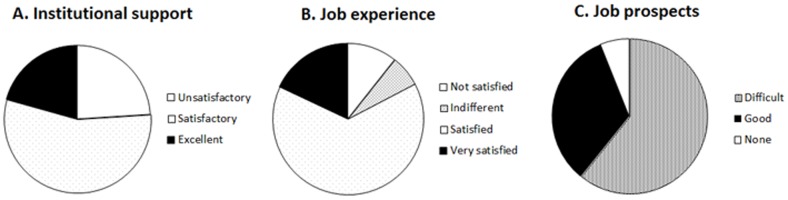
Summary of PDRs' responses about their work environment and expectations. (A) Institutional support, (B) Job experience, and (C) Job prospects.

PDRs rated the support they received from their research institution (e.g. staff development courses, teaching training). The level of institutional support did not vary significantly with productivity (*F*<1). Overall, the level of institutional support was considered appropriate (Figure 1A). However, PDRs with children tended to find the level of institutional support more unsatisfactory than PDRs without children (χ^2^ (2, *N* = 149) = 6.69, *p*<.04).

#### Job satisfaction

Contrary to expected, job satisfaction did not vary with productivity (*F*(4, 143) = 2.02, *p* = .09). The majority of the PDRs (64%) were satisfied with their job, 19% were very satisfied and 17% were not satisfied or were indifferent to it (Figure 1B).

#### Job prospects

There were three questions related to PDRs' job prospects (see Supporting Information). Most PDRs thought it would be difficult to obtain a job in academia or in the private sector (67%), whereas the remaining PDRs were more confident they would find a tenured job and relied on the support received from their institution. The research output did not vary with PDRs' perceived job prospects (*F*(3, 142) = 1.32, *p* = .27) (Figure 1C). Further detailed studies are needed to identify effective type of support to enhance their job prospects.

#### Work allocation (research, teaching, administration)

PDRs were asked to estimate the proportion of their working time allocated to research, teaching and administrative duties. Full-time PDRs allocated 88% of their time to research, 5% to teaching and 7% to administrative/other duties, which was similar to part-time PDRs (research  = 82%, teaching  = 8%, administrative duties  = 10%).

The time PDRs allocated to administrative, research and teaching duties was not correlated to their productivity. However, there was a correlation between time dedicated to administrative duties and gender (*r*(187) = .16, *p* = .03), whereby males (*Mean*  = 9%, *SE*  = 2) spent a higher proportion of time on such duties than female (*Mean*  = 5%, *SE*  = 1). The question as to whether this early imbalance in administrative duties was related to the gender discrepancies found later in academic tenured positions remains open.

## Discussion

This study explored, for the first time, links between family and work environments and the research output of PDRs based in the UK. The government's Science and Innovation Investment Framework put science at the heart of economic progress, but a successful implementation of such a strategy demands a workforce with a high level of expertise. Therefore, it is fundamental not only to foster but also to increase the retention of highly skilled and trained scholars in research institutions in the UK, and this may be facilitated by high quality postdoctoral training. Academics with PDR training have been shown to be more engaged in international academic exchanges than their peers without postdoctoral experience [30], [31]. Furthermore, as the title of a study points out “a positive postdoctoral experience is related to quality supervision and career mentoring, collaborations, networking and a nurturing research environment” [11].

Anecdotally, many scientists believe that the output in research centres is higher than in universities, which was counter to our results. The findings showed that the research place was associated with the output, which tended to be slightly higher in universities than in research centres. The causes for this difference are not clear. It could be linked to the use of more complex equipment and techniques in highly specialized research centres, which could have led to more time-consuming experiments and, therefore, fewer publications in the same given period. In line with such possibility, results from the first two years of employment showed that PDRs in Life Science—who were more likely to be in research centres—had an output of 0.22 peer-reviewed articles/year, whereas in Social Sciences the output was 0.82. Alternatively, research groups in research centres tend to be large and so it is their combined output, but each individual in the group may have a relatively small output. Ideally, one would need to analyse PDRs' output in terms of number of co-authors as well as the complexity of experimental setups and paradigms used, which was not possible in an anonymous study. Furthermore, the output in Social Sciences would need to take into account other forms of high quality research output [32].

There were no statistically significant differences in output according to nationality in terms of British and non-British PDRs, but a larger sample of non-European PDRs is needed for further comparisons.

Interesting results, or rather an interesting lack of results, emerged when output was analysed in relation to the presence or absence of children and PDRs' gender. Overall, male and female PDRs did not differ in productivity. Contrary to what was expected, PDRs with children tended to publish as much as PDRs without children, but they also tended to stay longer in postdoctoral employment. The output of male and females PDRs without children was identical, whereas male PDRs with children tended to publish slightly more than female PDRs with children. Although this result was in line with previous studies reporting some of the difficulties faced by female PDRs with children [17], [33], the difference in this study was not statistically significant. While the common perception is that children may affect the working lives of academics—particularly mothers—by reducing the time available for academic work, it is possible that the presence of children in the after-work environment has a positive effect by helping parents in demanding jobs to “unwind” and stop job-related rumination [34]. Likewise, worries about the costs of raising children could have led some parents to try to secure more publications to increase their chances of a tenured position. It is difficult to draw wide-range conclusions with this exploratory study; there is an urgent need for large-scale studies to confirm the effect of children on the PDRs' research output.

A strong correlation among family size, birth order and academic achievement has been reported in earlier studies [21]–[24]. However, in this study the research output was linked neither to the PDR's number of siblings nor to their birth order. This may be because PDR productivity is determined by factors different to academic achievement early in life (by definition, all PDRs are high academic achievers). Interestingly, the output was positively correlated to the educational level of their fathers (i.e. the more educated the fathers were, the higher the output), but not of their mothers.

The findings also suggested that the work environment did not affect PDRs' output directly, although institutional support might have modulated the level of job satisfaction [35], [36]. The level of PDRs' satisfaction with their current jobs was rather high: only 17% were dissatisfied with or indifferent to their current job. However, almost a quarter of the PDRs were not happy with the support provided by their institution, and a high percentage (67%) thought their job prospects were poor, which provides cause for some concern, also raised in studies elsewhere [37], [38]. This may reflect a general concern among younger employees in all professions about their job prospects [39], though a careful comparison with other professions is not possible at this stage. Whilst currently such dissatisfaction did not appear to impinge on PDRs' research output, there is no guarantee that such a state of affairs will continue, due to changes in the structuring and funding of the HE environment since this data was collected. Together with developments in the broader economy, such changes may affect how subsequent generations of researchers are both attracted and funded in the UK.

This study had some limitations. In addition to the relatively small sample set if one wishes to maximize avoidance of Type II error, the present findings stem from voluntary participation, i.e. PDRs filling in the questionnaire might have been particularly happy or particularly unhappy with their PDR experience. On the other hand, its compulsory completion would have other limitations. However, there is little data currently available on the postdoctoral experience in the UK. This research focussed solely on PDRs and may not reflect the experience of UK academics more generally, since postdoctoral researchers are likely to be unevenly distributed across disciplines: postdoctoral work is more likely in the sciences than the humanities or some areas of social sciences and across work places (a higher proportion of researchers in research centres are likely to be PDRs compared to those in the universities).

This snapshot is a useful step towards a better understanding of PDRs work/training experience in the country, and we consider that this study is a helpful first step towards describing the UK postdoctoral experience and highlighting fruitful avenues for future research. It contributes empirical data demonstrating how PDRs felt about their work and identifies some of the factors which may be associated with their output—a significant determinant of whether they will achieve a tenure-track post. This is also an attempt to entice other researchers to investigate the issues raised in this study in more depth, and it suggests that PDRs' output, employment conditions, and job prospects need to be closely monitored—all the more so given the importance of PDRs' work in research and development for the UK economy.

## Supporting Information

Table S1 **Universities and specialized, independent research centres represented in this study.** The number of participants from each institution is given in the parenthesis.
(DOCX)

Table S2 **Some of the questions used in this study.** The questions are grouped by core topic. Questions related to the different questionnaires (*N* = 5) were removed. PDR refers to postdoctoral researcher (also referred to as “postdoc”).
(DOCX)
