# Supplementary material for: Postdoctoral Researchers in the UK: A Snapshot at Factors Affecting Their Research Output
Source: PLoS One. 2014 Apr 4;9(4):e93890. doi: 10.1371/journal.pone.0093890 (PMC3976345; doi:10.1371/journal.pone.0093890)
Supplement: Table S1 — Universities and specialized, independent research centres represented in this study. The number of participants from each institution is given in the parenthesis. (DOCX) [file pone.0093890.s001.docx]

**Table S1**. Universities and specialized, independent research centres represented in this study. The number of participants from each institution is given in the parenthesis.

| 1 U. Cambridge (*N* = 53)  2 U. Bristol (*N* = 8)  3 U. College London (*N* = 8)  4 U. Exeter (*N* = 7)  5 Cardiff U. (*N* = 5)  6 King's College U. (*N* = 5)  7 U. Glasgow (*N* = 4)  8 U. Loughborough (*N* = 4)  9 U. Manchester (*N* = 4)  10 Queen Mary U. London (*N* = 4)  11 Bangor U. (*N* = 3)  12 City U. (*N* = 3)  13 U. Aberdeen (*N* = 2)  14 U. Birmingham (*N* = 2)  15 U. Leicester (*N* = 2)  16 U. Oxford (*N* = 2)  17 Stirling U. (*N* = 2)  18 Swansea U. (*N* = 2)  19 U. Bath (*N* = 1)  20 Birkbeck College (*N* = 1)  21 U. Bradford (*N* = 1)  22 Brunel U. (*N* = 1)  23 U. Central Lancashire (*N* = 1)  24 Coventry U. (*N* = 1)  25 Durham U. (*N* = 1) | 26 U. East Anglia (*N* = 1)  27 U. Edinburgh (*N* = 1)  28 U. Essex (*N* = 1)  29 U. Hull (*N* = 1)  30 Imperial College U. London (*N* = 1)  31 Kingston U. (*N* = 1)  32 U. Leeds (*N* = 1)  33 Liverpool U. (*N* = 1)  34 Manchester Metropolitan U. (*N* = 1)  35 Newcastle U. (*N* = 1)  36 U. Nottingham (*N* = 1)  37 U. Reading (*N* = 1)  38 Royal Holloway U. London (*N* = 1)  39 U. Sheffield (*N* = 1)  40 Sheffield Hallam U. (*N* = 1)  41 U. Southampton (*N* = 1)  42 U. St Andrews (*N* = 1)  43 U. Sussex (*N* = 1)  44 U. Warwick (*N* = 1)  45 U. York (*N* = 1)  **Research centres**  1 Cancer Research UK (*N* = 38)  2 Medical Research Council (*N* = 5)  3 Babraham Institute (*N* = 2)  4 European Bioinformatics Institute (*N* = 1) |
| --- | --- |

U. = university
