# Supplementary material for: Postdoctoral Researchers in the UK: A Snapshot at Factors Affecting Their Research Output
Source: PLoS One. 2014 Apr 4;9(4):e93890. doi: 10.1371/journal.pone.0093890 (PMC3976345; doi:10.1371/journal.pone.0093890)
Supplement: Table S2 — Some of the questions used in this study. The questions are grouped by core topic. Questions related to the different questionnaires (N = 5) were removed. PDR refers to postdoctoral researcher (also referred to as “postdoc”). (DOCX) [file pone.0093890.s002.docx]

**Table S2.** Some of the questions used in this study. The questions are grouped by core topic. Questions related to the different questionnaires (*N* = 5) were removed. PDR refers to postdoctoral researcher (also referred to as “postdoc”).

| \| 1. Gender 2. Age 3. Nationality 4. Number of siblings (including step- and foster siblings) 5. Birth order 6. Mother's education (latest degree obtained) 7. Father's education (latest degree obtained) 8. Present of own children. If yes, how many 9. Area of your first university degree 10. Other qualifications have you obtained and completion year (MA, MSc, PhD, etc) 11. Current research institution (and address) 12. Current research area 13. Title of your current job 14. Years working as a research assistant, associate, fellow, or any other post-doctoral research position 15. Full-time or part-time PDR 16. Years as a PDR 17. What percentage of your working time do dedicate to:  - *research (%)* - *teaching (%)* - *administrative duties (%)*  1. Number of peer reviewed articles (as a PDR; add ZERO if no publications are yet available) 2. Number of peer reviewed abstracts or conference talks (as PDR) 3. Number of book(s) or book chapter(s) (as PDR) 4. Other publications (as PDR) 5. Total number of peer reviewed articles per year of PDR (calculated by researchers, not PDRs) 6. Start and finish times of a normal working day 7. Level of your satisfaction with your PDR job (“How happy are you with your postdoctoral experience so far?”):    1. Very satisfied    2. Fairly satisfied    3. Slightly satisfied    4. Not satisfied at all    5. Indifferent 8. Level of institutional support for PDRs at your research institution (“What is your assessment of the quality of faculty support for your work?”)    1. Excellent    2. Satisfactory    3. Unsatisfactory    4. Other (please specify) 9. Awareness of retention plans for PDRs at your research institution (“Does your institution have recruitment policies that reflect a sound plan for retaining postdocs who excelled in their work?”)    1. Yes, the policies reflect a sound plan.    2. There are policies, but they don't seem tied to a plan.    3. There are no such policies in place to retain bright postdocs.    4. Other (please specify) 10. Job prospects at current research institution (“Does your organization enhance or hinder your ability to become a successful faculty member?”)     1. The organization's structure has no effect on my ability to be successful.     2. The organization's structure does not help postdocs to find work placements.     3. The organization helps me to be successful     4. The organization's structure hinders my ability to be successful.     5. Other (please specify) 11. Job prospects in general (“How do you see your job prospects once you finish your current postdoc contract?”)     1. Good possibilities in academia/public/private sector.     2. Difficult job market, but I will find a job in academia/public/private sector.     3. No job market in academia/public/private sector. I will have to give up a career in Science.     4. Other (please specify) \| \| --- \| \|  \| |
| --- | --- | --- |
